# Ketogenic Diet as Medical Prescription in Women with Polycystic Ovary Syndrome (PCOS)

**DOI:** 10.1007/s13668-023-00456-1

**Published:** 2023-01-25

**Authors:** Luigi Barrea, Ludovica Verde, Elisabetta Camajani, Simona Cernea, Evelyn Frias-Toral, Dilusha Lamabadusuriya, Florencia Ceriani, Silvia Savastano, Annamaria Colao, Giovanna Muscogiuri

**Affiliations:** 1 Dipartimento di Scienze Umanistiche, Università Telematica Pegaso, Via Porzio, Centro isola F2, 80143 Naples, Italy; 2 Centro Italiano per la cura e il Benessere del paziente con Obesità (C.I.B.O), Department of Clinical Medicine and Surgery, Endocrinology Unit, University Medical School of Naples, Via Sergio Pansini 5, 80131 Naples, Italy; 3 Department of Human Sciences and Promotion of the Quality of Life, San Raffaele Open University, 00166 Rome, Italy; 4 PhD Program in Endocrinological Sciences, Department of Experimental Medicine, University of Rome “La Sapienza”, Piazzale Aldo Moro, 5, 00185 Rome, Italy; 5 George Emil Palade University of Medicine, Pharmacy, Science, and Technology of Târgu Mures/Internal Medicine I, Târgu Mureş, Romania; 6 Diabetes, Nutrition and Metabolic Diseases Outpatient Unit, Emergency County Clinical Hospital, Târgu Mureş, Romania; 7 Universidad Católica Santiago de Guayaquil, Guayaquil, Ecuador; 8 University Hospital General Sir John Kotelawala Defence University, Boralesgamuwa, Sri Lanka; 9 Nutrition School, Universidad de la Republica (UdelaR), Montevideo, Uruguay; 10 Dipartimento di Medicina Clinica e Chirurgia, Unit of Endocrinology, Federico II University Medical School of Naples, Via Sergio Pansini 5, 80131 Naples, Italy; 11 Cattedra Unesco “Educazione alla salute e allo sviluppo sostenibile”, Federico II University, Naples, Italy; 12 Department of Public Health, University of Naples Federico II, Naples, Italy

**Keywords:** Polycystic ovary syndrome, Very low-calorie ketogenic diet, Diet, Nutrition, Nutritionist

## Abstract

**Purpose of Review:**

The polycystic ovary syndrome (PCOS) is an endocrine dysfunction associated with a myriad of metabolic disorders and high rate of infertility. In order to aid its management, several lifestyle/dietary interventions have been evaluated. Very low-calorie ketogenic diet (VLCKD) is rapidly showing promising benefits not only in obesity but also in the treatment of other metabolic diseases. The main objective of this review is to assess the scientific evidence in support of this dietary pattern as an effective measure for treating PCOS and the metabolic disorders associated with it.

**Recent Findings:**

Preliminary data suggested significant improvements in body weight and composition, metabolic profile (glucose, serum insulin, triglycerides, total and low-density lipoprotein cholesterol), and insulin resistance following VLCKD. However, the evidence is still scarce and needs to be more substantiated.

**Summary:**

Weight reduction in women with PCOS has been shown to improve metabolic derangements and body composition, but there is no consensus on the ideal dietary pattern or macronutrient composition. There is some evidence supporting the possible role of the Mediterranean diet in improving infertility (along with other well-known metabolic benefits) in women with PCOS. Of note, VLCKD might be considered a potential intervention for the short-term treatment of PCOS, but it must be prescribed and carefully guided by professionals.

## Introduction

Polycystic ovary syndrome (PCOS) is a lifelong endocrine dysfunction affecting 10 to 15% of women worldwide [1, 2]. According to the Rotterdam criteria, it is diagnosed when the person presents at least two of the three following criteria: ovarian cysts assessed by ultrasound examination; clinical hyperandrogenism with high circulating androgens levels; and oligo-amenorrhea with oligo-anovulation [2]. Due to the variety of clinical manifestations, in 2012, the National Institute of Health (NIH) consensus panel proposed the phenotypic approach to classify PCOS [3]. In brief, phenotype A (full-blown syndrome PCOS) includes hyperandrogenism (clinical or biochemical), ovulatory dysfunction, and polycystic ovaries. Phenotype B includes hyperandrogenism and ovulatory dysfunction. Phenotype C (ovulatory) includes hyperandrogenism and polycystic ovaries. Phenotype D (non-hyperandrogenic PCOS) includes ovulatory dysfunction and polycystic ovaries [3].

This pathology is responsible for various complications, including infertility in 40% of affected women [4]. Also, PCOS and adrenal incidentalomas have frequently been related to hyperinsulinemia and insulin resistance (IR) [5]. Likewise, it has been associated with metabolic disorders such as glucose intolerance, type 2 diabetes mellitus (T2D), hepatic steatosis, and hypertension [6]. Barrea et al. reported in 2018 that high carbohydrate intake and low-grade inflammation influence the development of IR and hyperandrogenism, thus influencing the pathophysiology of PCOS [7].

On the other hand, it should be noted that both obesity and obesity-related low-grade inflammation are common in PCOS [8, 9]. Considering this, the importance of nutrition in preventing and treating PCOS is evident. Body weight control is recommended, as a fundamental strategy for its treatment, although this is not always easily achieved [1, 4, 10]. However, it should be highlighted that there is still no consensus on the best dietary pattern to follow in order to effectively lose weight in women with PCOS [11]. In their review on nutritional management in women with PCOS, Faghfoori et al. emphasize the importance of adherence to a hypocaloric diet to achieve weight loss or maintain a healthy weight and highlight the need to limit the intake of simple sugars, refined carbohydrates, and saturated and trans-fatty acids [12].

The role of several eating patterns in the management of PCOS has already been investigated: the low saturated fat diet, the low glycemic index (GI) diet, and the ketogenic Mediterranean diet with phytoextracts (KEMEPHY) [11]. In this context, very low-calorie ketogenic diet (VLCKD) has been proposed as a possible approach to obesity, and the consensus of the Italian Society of Endocrinology (SIE) makes a weak recommendation for VLCKD in PCOS-associated obesity [13]. Ninety percent of the calories in this diet come from fats, and the remaining 10% are from carbohydrates and proteins. Usually, this type of diet is designed and divided into three stages. The first one is called active, then reeducation, and finally the maintenance phase. VLCKD is associated with a rapid weight loss, while the fat-free mass, which plays an important role in glucose metabolism, is preserved [13].

The main objective of this review is to assess the scientific evidence in support of this dietary pattern as an effective measure for treating PCOS and the metabolic disorders associated with it.

## Nutritional Approach to PCOS

PCOS has previously been associated with changes in body composition and carbohydrate metabolism disorders [12]. Moreover, in women with PCOS and obesity, significant alterations in carbohydrate, lipid, and amino acid metabolism with specific metabolomic signatures have been identified (e.g., decrease in citric and lactic acid levels, lysophosphatidylcholines, and glycerolphosphocholine and increase in free fatty acids (carnitine, adipic acid, linoleic acid, oleic acid) etc.) [14].

The literature shows that obesity, IR, and compensatory hyperinsulinemia coexist low-grade chronic inflammation within this syndrome [1]. As already mentioned, women with PCOS often have IR. Therefore, among the main goals of medical nutrition therapy for women with PCOS, reduction of IR and improvement of reproductive function are the most important. Reducing 5 to 10% of the usual weight can improve reproductive function [15]. This objective may not be achieved by weight loss alone. Hence, reducing the consumption of foods rich in fatty acids and high glycemic index (GI) and increasing the intake of omega 3 polyunsaturated fatty acids, vitamin D, and foods rich in chromium might bring additional benefit [12]. Thus, eating habits and dietary patterns might play a pivotal role in the prevention and treatment of PCOS.

The effect of diet in managing IR in PCOS is a controversial topic. Therefore, many studies have been undertaken to explain if certain diets help improve the metabolic control of women with PCOS. The meta-analysis by Shang et al. evaluated 19 clinical trials (1193 patients) and showed that dietary programs with restricted diets significantly improved IR, fasting glucose and body composition [16]. Therefore, offering patients professional advice regarding specific and individualized nutrition plans may positively affect PCOS control.

Different dietary patterns have been evaluated for the therapy of PCOS. More than 15 years ago, Farshchi et al. affirmed that the best way to treat and improve endocrine features, reproductive function, and cardiometabolic risk profile in PCOS, even without weight loss, is the diet and exercise approach [17].

### Mediterranean Diet

One of the main dietary interventions evaluated in women with PCOS is the Mediterranean diet (MD), which has proven to have anti-inflammatory effect and help decrease body weight [11, 18]. The MD is based on the regular consumption of fiber, vitamins, antioxidants, as well as unsaturated fats, carbohydrates with a low GI, and a moderate intake of animal protein [1]. The anti-inflammatory effect of this diet is attributed to the microbiota-derived production of short-chain fatty acids induced by dietary fiber and the high intake of both omega 3 polyunsaturated fatty acids and antioxidants [11]. The case-controlled study by Barrea et al. assessed the body composition, dietary intake, and adherence to the MD and their link with PCOS clinical severity in a cohort of 112 women with PCOS that had not received any PCOS treatment compared with a control group of 112 healthy women matched for age and body mass index (BMI). Diet adherence was assessed with a seven-day food record and the body composition by bioelectrical impedance analysis. C reactive protein (CRP) was measured as an indicator of inflammation, while the severity of PCOS was assessed with the use of Ferriman-Gallwey score. The results showed that women with PCOS had higher testosterone and fasting glucose levels, homeostatic model assessment for insulin resistance (HOMA-IR), and Ferriman-Gallwey scores, more fat mass and lower phase angle. The authors also found that dietary practice intake was different in the two groups, as PCOS women had lower ingestion of nuts, legumes, fish, and extra-virgin olive oil, essential components of the MD, compared to the control group. Testosterone levels were associated with higher CRP levels, as well as with dietary consumption of simple carbohydrates, saturated fatty acids, and omega 6 polyunsaturated fatty acids. The authors concluded that there is preliminary evidence to support the role of the MD diet in the management of PCOS, to reduce inflammatory status, IR, and hyperandrogenemia [11].

### Ketogenic Diet

The ketogenic diet (KD) is based on a low carbohydrates intake, high levels of fat (more than 70% of calories consumed), with avoidance of excess protein, which results in high production of ketones (mainly acetoacetate and β-hydroxybutyrate) and nutritional ketosis [19]. A noteworthy effect of KD for PCOS is adenosine monophosphate-activated protein kinase (AMPK) and silent mating type information regulation 2 homologue 1 (SIRT1) activation, even if it is not a caloric deprivation diet [20, 21•]. Once activated, SIRT1 and AMPK positively affect glucose homeostasis and improve insulin sensitivity [21, 22].

KD has been commonly used in the treatment of nervous system diseases, but research indicated that it could be a valid strategy for the treatment of metabolic diseases, T2D, obesity, and non-alcoholic fatty liver disease (NAFLD) [23]. This type of diet decreases postprandial insulin secretion but, in turn, reverses IR by inducing weight loss and fat mass loss. Paoli et al. investigated the effects of the KD in women of childbearing age with a diagnosis of PCOS [21•]. In this study, fourteen overweight women diagnosed with PCOS were put on a KEMEPHY for 12 weeks. The authors observed a significant decrease in blood glucose and insulin levels and a significant improvement in HOMA-IR. Likewise, the luteinizing hormone (LH)/follicle stimulating hormone (FSH) ratio, total and free testosterone, and blood levels of dehydroepiandrosterone sulfate (DHEAS) were significantly reduced [21•]. On the other hand, estradiol, progesterone, and sex hormone binding globulin (SHBG) increased [12]. The authors concluded that the KD can be considered for the non-pharmacological treatment of PCOS, but more studies with extended treatment periods should be carried out to verify the effects [21•].

Since PCOS is a chronic disease, and the KD has not been evaluated on long-term, a VLCKD has been proposed as an alternative. In 2016 the Italian Association of Dietetics and Clinical Nutrition and the Italian Society of Obesity proposed the VLCKD as a therapeutic option for drug-resistant epilepsy, NAFLD, and obesity associated with comorbidities, as well as for weight loss before bariatric surgery [24]. This diet provides ≤ 800 kcal per day and can be carried out with conventional foods or synthetic formulas (shakes, soups or bars, or a combination of both) [24]. Andersen et al. carried out a dietary intervention study in 9 women with PCOS and obesity aged 22 to 39 years, who followed a high-protein, very low-calorie diet, and after 4 weeks of treatment, significant reductions in fasting glucose levels and insulin were observed, as well as a significant increase in insulin sensitivity [25].

### Low-Glycemic Index Diets

These diets are characterized by most carbohydrates from low GI sources [26]. Diets with a high GI might directly impact IR through their effect on blood glucose, free fatty acids, and the secretion of counterregulatory hormones. Some data shows that GI of is more important than the total carbohydrate intake [19]. This type of diet has become popular in the treatment of PCOS. Apparently, women with PCOS have a lower glutathione peroxidase concentration than healthy women [27]. Thus, it has been suggested that the low GI diet decreases inflammation in women with PCOS by increasing uric acid concentration and glutathione peroxidase activity [27].

A recent meta-analysis of eight studies (including 412 women with PCOS and obesity or overweight) indicated that low GI diets improved the clinical and biochemical features of PCOS (hirsutism, IR, hormonal profile, infertility) and emotional health [28••].

### Pulse-Based Diet

Legumes such as lentils, peas, beans, and chickpeas are rich in fiber, contain complex carbohydrates with a low GI and high-quality proteins, are low in fats, and are an essential source of micronutrients [29]. In healthy subjects, this diet has been shown to prevent or reduce IR [29]. Kazemi et al. compared the effects of a low GI pulse-based diet to the Therapeutic Lifestyle Changes (TLC) diet on cardiometabolic measures in women with PCOS [30]. The group of women on the pulse-based diet had a greater decrease in the total area under the curve (AUC) for insulin response to a 75-g oral glucose tolerance test than the TLC group and improved lipid profile, indicating that a legume-based diet may be more effective in improving cardiometabolic risk factors in women with PCOS [30]. The same group subsequently compared the two interventions with regard to their effect on ovarian morphology, hyperandrogenism, and menstrual irregularity in women PCOS [31]. Both interventions improved ovarian dysmorphology (follicle numbers per ovary, ovarian volume), hyperandrogenism, and menstrual irregularity, with some of the benefits maintained 6 months postintervention [31].

### Dietary Approaches to Stop Hypertension

The Dietary Approaches to Stop Hypertension (DASH) is a dietary pattern high in carbohydrates and fibers, magnesium, potassium, and calcium (and other micronutrients); low in fats (mainly saturated fat); and moderate in proteins, mainly originating from fruits, vegetables, whole grains, nuts, legumes, and low-fat/skim dairy products, with reduced content of red and processed meats, refined grains, and sweets [32]. The DASH diet was designed primarily for blood pressure control [32]. According to the literature, a high dietary fiber intake contributes to higher insulin and glucose responses and is inversely correlated with fasting insulin, HOMA-IR, and the Matsuda insulin index [33].

Shang et al. conducted a systematic review in eight databases in 2019 to evaluate whether diet could reduce IR in women with PCOS [16]. The authors observed that dietary changes were significantly related to decreased IR and improved body composition in women with PCOS and identified that the DASH diet and calorie-restricted diets were most effective in improving insulin sensitivity in PCOS [16]. In fact, several studies demonstrated that DASH diet had beneficial effect on markers of IR, inflammation, oxidative stress, and on hormonal profile (reduced androgens, anti-Müllerian hormone) [34–37].

### Other Nutritional Interventions

A number of additional nutritional interventions have been evaluated for their effect on PCOS. A literature review by Muscogiuri et al. concluded that myo- and D-chiro-inositol isomers might be effective in improving metabolic profiles and ovarian function and in patients with PCOS, but more data (mainly longitudinal, interventional studies) is needed [38].

Jamilian et al. performed a RCT to evaluate the effects of probiotic and selenium co-supplementation in a group of 60 women with PCOS [39]. They were randomly to receive either 8 × 10^9^ CFU/day probiotic containing *Lactobacillus acidophilus*, *Lactobacillus reuteri*, *Lactobacillus fermentum*, and *Bifidobacterium bifidum* plus 200 μg/day selenium supplements or placebo for 12 weeks. The authors found that the supplemented group significantly improved the testosterone levels, hirsutism, high-sensitivity CRP levels, and the oxidative status measured by plasma malondialdehyde levels. They also showed significant differences in increasing total antioxidant status and glutathione in plasma compared to the placebo group [39].

It is evident that the nutritional approach in treating PCOS is essential, whether in controlling body weight, IR, or treating associated comorbidities through different strategies. It seems essential to continue carrying out studies that generate evidence on which dietary pattern is most indicated for managing this condition.

## PCOS and VLCKD

Dietary approaches to PCOS have been discussed extensively, but data is limited only to the short term, leaving an unanswered question of whether there is a superior protocol for this condition. PCOS has been associated with obesity, weight changes, cardiovascular diseases, and carbohydrate metabolism alterations, such as IR and insulin secretion [5]. Therefore, hyperinsulinemia impairs the production of sex hormones [40].

There is an urgent need to implement an anti-inflammatory dietary intervention to treat PCOS due to the substantial role of chronic inflammation in the pathogenesis of numerous chronic diseases and related complications caused by PCOS in women across their entire life course [1, 7, 11]. From the papers analyzed in this respect, weight management and caloric restriction are pillars of IR treatment. Reducing weight in women with PCOS can improve IR, hypertension, dyslipidemia, T2D, and other related morbidities, but ideal macronutrient distribution has not been standardized yet [1, 7, 11].

In a study conducted by Cincione et al., it was demonstrated that KD improved anthropometric parameters, such as body weight, waist circumference, and fat mass, but also biochemical parameters, such as LH, FSH, SHBG, and HOMA-IR [41]. The authors demonstrated a major improvement in IR, in just 6 weeks, a reduction in fat mass, leading to a reduction in acyclic estrogen production resulting from the aromatization in adipose tissue of androgenic excess, with an improvement in the LH/FSH ratio [41]. Very recently, a retrospective study assessed the effect of the VLCKD on markers suggested to be predictive of metabolic and ovulatory dysfunction [42]. Twenty-five women with obesity and PCOS underwent VLCKD for 12 weeks. After the nutritional intervention, women showed significant decrease in serum levels of anti-Müllerian hormone and significant increase in progesterone and SHBG. Therefore, the authors concluded that VLCKD could also benefit ovarian reserve and luteal function [42].

Thus, it seems that a ketogenic approach could be effective in targeting various clinical manifestation of PCOS and so to be a useful tool for also the different phenotypes of PCOS.

It should be noted that some women with PCOS suffer from IR, but not obesity [1]. Muscogiuri et al. evaluated the association of vitamin D levels with insulin sensitivity, body composition, and endocrine parameters in 38 women with PCOS and concluded that vitamin D levels are significantly influenced by the degree of adiposity [43]. Also, they stated that this deficiency might worsen IR in women with PCOS [43]. Hence, weight loss and decreased fat mass might restore regular vitamin D levels and insulin sensitivity. As reported by Buscemi et al., vitamin D blood levels were significantly lower in subjects with obesity, probably due to its uptake by the adipose tissue [44]. In their study, they evaluated the change in vitamin D concentrations in 31 subjects with obesity before and after dietary treatment with VLCKD. Subjects with obesity had lower vitamin D levels, and at the end of the 10–12 weeks dietary intervention, the increase in vitamin D levels correlated with the reduction in body weight and especially fat mass [44]. Thus, this study supports the hypothesis that vitamin D is stored in adipose tissue and released after weight loss. Furthermore, 25(OH)D supplementation might reduce IR and metabolic syndrome in PCOS [45].

For lean women with PCOS, the focus is on weight maintenance [1]. Regardless of adiposity levels, most patients with PCOS have high serum insulin and IR obesity. Lifestyle modifications such as dietary interventions, improved sleep patterns, and physical activity have ameliorated IR and hormone profiles in subjects with obesity [1, 46].

A VLCKD has been proposed as an attractive nutritional strategy for treating obesity [13, 47]. There are three stages of this protocol: active, reeducation, and maintenance. These stages are in fact further sub-divided into five steps: step 1 is characterized by high-biological-value proteins and based on meals replacement with low GI vegetables; in step 2, one or two protein servings are replaced by natural protein meal such as meat/egg/fish either at lunch or dinner. These are part of the first active step of VLCKD, which is characterized by a very low-calorie intake (650–800 kcal/day), low in carbohydrates (< 30 g daily from vegetables), and lipids (only 20 g per day, derived also from olive oil). The daily amount of high-biological-value proteins usually ranges between 1.2 and 1.5 g/Kg ideal body weight in order to preserve lean mass. Also, a supplementation with vitamins and minerals (K, Na, Mg, Ca, and omega-3 fatty acids) is included in the plan. The active stage can be prolonged for 8–12 weeks. The reintroduction phase is based on the gradual introduction of different food groups and the stepwise increase of the average daily calorie intake. This phase also comprises two steps: step 3 is based on a calorie content of about 900–1200 kcal, and low GI foods, including dairy products and legumes, are reintroduced; step 4 is based on a calorie content of about 1250–1500 kcal and fruit, and low GI cereals are reintroduced. In the last phase, the maintenance phase (step 5), a balanced low-calorie diet is set, following the MD. This last phase, through the acquisition of correct eating habits, is crucial for maintaining the long-term results. The maintenance stage allows 1500 to 2000 kcal/day, depending on individual nutritional requirements, in order to maintain long-term weight loss and promote a healthy lifestyle. The recommendation of VLCKD should be limited to specific patients and under supervision because of its contraindications and side effects (Tables 1 and 2) [13, 47, 48].

**Table 1:** Contraindications to VLCKD according to ADI and SIE

**ADI**	**SIE**
Pregnancy and lactation History of mental and behavioral problems Alcohol and other substances abuse Hepatic or renal failure Type 1 diabetes Porphyria Unstable angina History of recently myocardial infarction	Type 1 diabetes mellitus Autoimmune diabetes in adults β-cell failure in diabetes mellitus Use of SGLT2 inhibitors (risk for euglycemic diabetic ketoacidosis) Pregnancy and breastfeeding Kidney disease and failure Liver failure Cardiorespiratory failure Unstable angina History of recently myocardial infarction and stroke Cardiac arrhythmias Eating disorders, mental illnesses, substance abuse, and addictions Infections Elderly patients 48 h prior to elective surgery and perioperative period Body metabolism disorders

*ADI* Associazione Italiana di Dietetica e Nutrizione Clinica, *SIE* Società Italiana di Endocrinologia, *SGLT2* sodium/glucose cotransporter 2

**Table 2:** VLCKD side effects

**Short term**	**Long term**
Dehydration Transient hypoglycemia Transitory lethargy Halitosis Gastrointestinal: vomiting, nausea, diarrhea, constipation Hyperuricemia	Hypoproteinemia Hypocalcemia Lipid profile alterations Urolithiasis Gallstones Hair loss

*TG* triglycerides, *HDL* high-density lipoprotein, *LDL* low-density lipoprotein

A systematic review and meta-analysis reviewed the effects of the VLCKD on weight, body composition, and metabolic profile [47]. Four specific findings are worth mentioning, namely that (a) VLCKD was associated with significant body weight reduction accompanied by improvements in body composition, glucose and lipid parameters; (b) compared with other weight loss interventions, VLCKD had a greater impact on decreasing body weight and fat mass, waist circumference, total cholesterol, and triglycerides and on improving insulin sensitivity; (c) compared with other weight loss interventions, the blood glucose, HbA1c, and low-density lipoprotein (LDL) cholesterol reduction were similar; and (d) the side effects were mild and transient. Therefore, VLCKD was recommended as an effective dietary protocol for obesity treatment and associated comorbidities that need immediate weight loss. Also, long-term lifestyle change is suggested once target weight attainment is achieved [47].

A recent study that included 106 individuals (12 males and 94 females) with obesity showed that VLCKD had mild side effects that could be avoided and controlled by adequately following the indications and contraindications for this diet [48]. The study findings proved that VLCKD is an effective and safe diet for patients with obesity. The VLCKD pattern consisted of replacement meals and the three stages already explained in this article. Side effects were evaluated by questionnaires, physical examination, and laboratory assessment. The weight from baseline to the end of the ketogenic phase was reduced from 94.38 ± 17.34 kg to 87.29 ± 15.99 kg (*p* < 0.001) and BMI from 34.98 ± 5.43 kg/m^2^ to 32.35 ± 5.02 kg/m^2^ (*p* < 0.001). A significant drop in the waist and hip circumferences from 106.16 ± 14.20 cm to 99.24 ± 13.57 cm (*p* < 0.001) and 120.53 ± 10.81 cm to 115.91 ± 9.70 cm (*p* < 0.001), respectively, was also observed. Additionally, glucose decreased from 88.04 ± 8.95 mg/dL to 82.60 ± 10.08 mg/dL (*p* = 0.072), insulin from 17.35 mg/dL ± 13.83 mg/dL to 8.05 ± 5.48 mg/dL (*p* = 0.286), and HOMA-IR from 3.80 ± 2.79 to 1.74 ± 1.29 (*p* = 0.332) showing a slight improvement, but not statistically significant. It is important to emphasize that the VLCKD should be done under the supervision of healthcare professionals for long-lasting effects [48].

However, interventions of VLCKD for PCOS in the long term have not been published so far. The trial by Paoli et al. consisted of 12 weeks of intervention with 14 women with PCOS of childbearing age [21•]. Participants followed a modified KD known as the KEMEPHY diet, which is a Mediterranean isocaloric ketogenic protocol of 1600/1700 kcal/day with added phytoextracts. The KEMEPHY protocol consisted of green leafy vegetables, cruciferous and limited amounts of meat, eggs, and fish. Four high-protein supplements (19 g/portion) and liquid herbal extracts were added. At the end of 12 weeks, almost all anthropometric, biochemical, and hormonal variables improved. Specifically, an average weight loss of 9.43 kg was observed (from 81.19 ± 8.44 kg to 71.76 ± 6.66 kg; *p* < 0.001), along with a significant reduction (− 3.35) of BMI (from 28.84 ± 2.10 kg/m^2^ to 25.49 ± 1.69 kg/m^2^; *p* < 0.001). Glucose decreased from 5.10 ± 0.25 mmol/L to 4.64 ± 0.24 mmol/L (*p* < 0.001), serum insulin from 12.62 ± 0.48 μU/mL to 11.31 ± 0.60 μU/mL (*p* < 0.001), and consequently HOMA-IR from 2.85 ± 0.15 to 2.32 ± 0.13 (*p* < 0.001) [17]. In addition, significant reductions in triglycerides (from 2.31 ± 0.40 mmol/L to 1.87 ± 0.27 mmol/L; *p* < 0.008), total cholesterol (from 5.36 ± 0.36 mmol/L to 4.72 ± 0.33 mmol/L; *p* < 0.001) and LDL cholesterol (from 3.11 ± 0.60 mmol/L to 2.33 ± 0.17 mmol/L; *p* < 0.001), and increase in high-density lipoprotein cholesterol levels (from 1.79 ± 0.41 mmol/L to 2.02 ± 0.43 mmol/L; *p* < 0.001) were noted [21•].

More consistent evidence is still needed before a certain nutritional approach for the PCOS management is recommend. Before dietary intervention (ideally individualized) is implemented for PCOS clinical treatment, careful nutritional assessment is critical to determine the best strategy.

## Conclusions

The worldwide prevalence of PCOS is quite high among women of fertile age. Moreover, its consequences are significant enough to justify the evaluation of possible nutritional interventions. Although the most frequently PCOS is associated with obesity, there are also PCOS patients with normal weight. In case of overweight or obesity, the aim of intervention is to decrease body weight and maintain it within the normal range.

It has been postulated that dietary nutrients can directly influence metabolic management, inflammation, and oxidative stress. Several types of dietary patterns have been suggested to treat PCOS. Among them, the MD is an alternative that favors the control of the inflammatory state, IR, and hyperandrogenemia.

The importance of an approach through diet and physical activity has become clear. As the drug therapy has proven effective on short term, probably the only approach with sustainable effects is a combination of a personalized diet and an exercise routine.

**Fig. 1:**
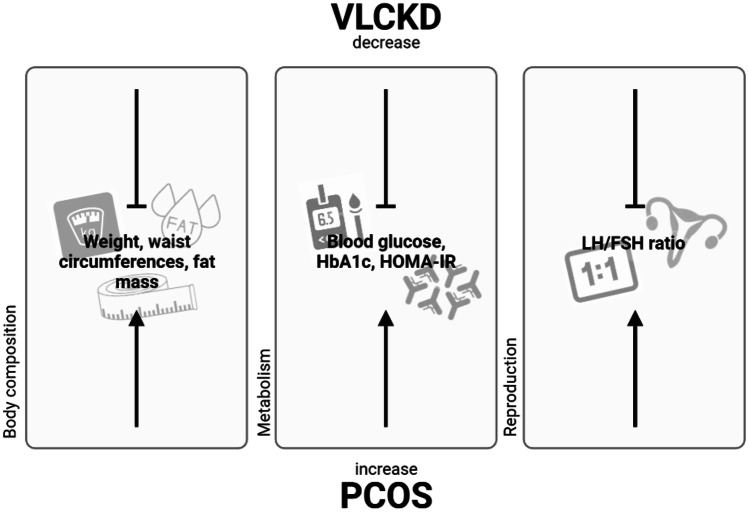
Metabolic and reproductive effects of VLCKD on PCOS. *VLCKD* very low-calorie ketogenic diet, *PCOS* polycystic ovary syndrome. ^⊥^ indicates a decrease, while ↑ indicates an increase

Data so far seem to indicate that VLCKD can be considered an effective dietary intervention for the short-term treatment of PCOS. It promotes rapid weight loss, with improvements in body composition and metabolic profile (waist circumferences, fat mass, blood glucose, HbA1c and HOMA-IR), and improvement of insulin sensitivity, fundamental aspects in the pathophysiology of PCOS (Fig. 1). Given its complexity, this dietary intervention must be recommended and guided by qualified professionals in the field. Also, it is essential to individualize the treatment and evaluate the contraindications and adverse effects. This type of diet is proposed in stages, the first being the most restrictive. It is essential to follow appropriately every step of the diet and achieve long-term weight loss maintenance and adherence to a healthy lifestyle.

It should be noted though that more studies on nutritional interventions for PCOS management are needed to provide more solid evidence for short- and long-term benefits, and long-lasting lifestyle changes.
