# Correction: Ketogenic Diet as Medical Prescription in Women with Polycystic Ovary Syndrome (PCOS)

**DOI:** 10.1007/s13668-023-00463-2

**Published:** 2023-02-16

**Authors:** Luigi Barrea, Ludovica Verde, Elisabetta Camajani, Simona Cernea, Evelyn Frias-Toral, Dilusha Lamabadusuriya, Florencia Ceriani, Silvia Savastano, Annamaria Colao, Giovanna Muscogiuri

**Affiliations:** 1 Dipartimento di Scienze Umanistiche, Università Telematica Pegaso, Via Porzio, Centro isola F2, 80143 Naples, Italy; 2 Centro Italiano per la cura e il Benessere del paziente con Obesità (C.I.B.O), Department of Clinical Medicine and Surgery, Endocrinology Unit, University Medical School of Naples, Via Sergio Pansini 5, 80131 Naples, Italy; 3 Department of Human Sciences and Promotion of the Quality of Life, San Raffaele Open University, 00166 Rome, Italy; 4 PhD Program in Endocrinological Sciences, Department of Experimental Medicine, University of Rome “La Sapienza”, Piazzale Aldo Moro, 5, 00185 Rome, Italy; 5 George Emil Palade University of Medicine, Pharmacy, Science, and Technology of Târgu Mures/Internal Medicine I, Târgu Mureş, Romania; 6 Diabetes, Nutrition and Metabolic Diseases Outpatient Unit, Emergency County Clinical Hospital, Târgu Mureş, Romania; 7 Universidad Católica Santiago de Guayaquil, Guayaquil, Ecuador; 8 University Hospital General Sir John Kotelawala Defence University, Boralesgamuwa, Sri Lanka; 9 Nutrition School, Universidad de la Republica (UdelaR), Montevideo, Uruguay; 10 Dipartimento di Medicina Clinica e Chirurgia, Unit of Endocrinology, Federico II University Medical School of Naples, Via Sergio Pansini 5, 80131 Naples, Italy; 11 Cattedra Unesco “Educazione alla salute e allo sviluppo sostenibile”, Federico II University, Naples, Italy; 12 Department of Public Health, University of Naples Federico II, Naples, Italy

**Correction to Current Nutrition Reports** 10.1007/s13668-023-00456-1

The author “Ludovica Verde“ should be retained as secondly listed in the authorgroup instead of last based on the accepted manuscript.

The original article has been corrected.

